# Determining the Feasibility and Usability of a Co-Designed Culturally Appropriate Conversational Agent (DESI-Heart) to Support Self-Care in People With Cardiovascular Diseases: Protocol for a Single-Arm Pilot Trial

**DOI:** 10.2196/78923

**Published:** 2025-11-28

**Authors:** Ann Tresa Sebastian, Paul Jansons, Ee Ling Ng, Samantha David, Ralph Maddison

**Affiliations:** 1 Faculty of Health Institute for Physical Activity and Nutrition Deakin University Burwood Australia; 2 Community Assessment and Response Team Monash Health Melbourne Australia

**Keywords:** conversational agent, self-care, cardiovascular diseases, culturally and linguistically diverse communities, digital health, technology, behavior change, methodological consideration, mobile phone

## Abstract

**Background:**

Cardiovascular diseases (CVDs) are a leading cause of death and disability worldwide. For people living with CVD, clinical guidelines recommend ongoing self-care such as symptom monitoring, medication adherence, and lifestyle modifications. However, many people struggle to engage in this due to the complexity of disease management, limited understanding, and a lack of cultural support. Conversational agents (CAs) offer a solution by providing artificial intelligence–driven, voice-based support that enables human-like communication. While many CAs and digital interventions are good for people with CVDs, they are for mainstream populations and overlook culturally and linguistically diverse communities.

**Objective:**

This study outlines the protocol for pilot testing the feasibility and usability of Diaspora Engaged Self-Care Intervention and Heart (DESI-Heart) program, to support self-care management among Indian diaspora populations with CVDs in Australia over an 8-week intervention period. The formative development of DESI-Heart is also described.

**Methods:**

We integrated the Double Diamond Model and the ecological validity model to develop our DESI-Heart program. First, we co-designed the program with end users, who identified 4 key goals for engagement with self-care through culturally and linguistically appropriate approaches. Based on these priorities and ideas, we developed specific goals, including (1) medication reminders, (2) daily exercise guidance, (3) diet buddy, and (4) guided meditation. Participants will access the DESI-Heart program through a web-based CA, available on smartphones, laptops, or PCs. Based on their preferred timing, individuals will receive links to access specific components of the program corresponding to each goal. These links will be sent to participants via SMS or email, depending on their preference. A single-arm prepost pilot trial (N=28) will be conducted to evaluate the feasibility and usability of the DESI-Heart program among Indian adults living in Australia with CVDs. The primary outcome will assess feasibility indicators, including recruitment, engagement, and usability, while secondary outcomes will examine changes in self-care behaviors and quality of life.

**Results:**

The DESI-Heart program received ethics approval in July 2024. Recruitment for the pilot trial is scheduled to begin in June 2025 and conclude by September 2025, with participant follow-up expected to be completed by the end of December 2025. All 28 participants have been recruited, and data analysis will be conducted once follow-up is finalized.

**Conclusions:**

We have co-designed and developed the DESI-Heart program, a culturally and linguistically appropriate self-care intervention aimed at supporting Indian adults with CVD living in Australia. The next step is to conduct a pilot study to assess the feasibility and usability of DESI-Heart, which will inform the design of a larger evaluation trial. DESI-Heart has the potential to complement existing health services by helping individuals with CVD manage their condition within the community, while acknowledging their cultural backgrounds and language preferences.

**International Registered Report Identifier (IRRID):**

PRR1-10.2196/78923

## Introduction

Cardiovascular disease (CVD) is the leading cause of death and disability worldwide, contributing to approximately 32% of the worldwide mortality rate [1]. In Australia, CVD contributes 19% to the total disease burden and accounts for approximately 27% of deaths [2]. Clinical guidelines recommend continuous self-care, including symptom monitoring, medication adherence, and lifestyle modification for effective management of these conditions [3]. Self-care is associated with reduced cardiac mortality, hospitalization and readmissions, and increased quality of life [4,5]. However, many individuals often struggle to incorporate and maintain consistent self-care due to complex regimens, limited health literacy, and lack of access to health care support [6,7].

The widespread availability and integration of digital technologies has enabled the development of innovative interventions to support self-care for people living with CVD in the community [8]. A systematic review and meta-analysis (n=9893) found that digital health services, such as mobile health interventions, played a key role in supporting self-care. These interventions were associated with a significant reduction in pain (Hedges g=−1.09; 95% CI −1.68 to −0.45) and disability (Hedges g=−0.77; 95% CI −1.59 to 0.05) [9]. Despite their potential, maintaining long-term engagement with digital interventions is challenging [10] due to users’ lack of health and digital literacy, privacy and security concerns, and the complexity of user interfaces [10-14].

Conversational agents (CA) have emerged as a promising solution to overcome some of these user and engagement barriers by leveraging artificial intelligence (AI)–based systems that use natural language processing, enabling human-like interactions with users [15,16]. CAs offer multiple advantages: (1) the ability to interpret human speech and respond to it in a personalized manner similar to human health care providers [17]; (2) the ability to integrate and connect with technologies (eg, sensors or wearable devices) to provide data driven self-care via a CA [18]; (4) reduce barriers around digital literacy for the user [19]; and (5) capacity to communicate in multiple languages and dialects [20]. However, most CA-delivered self-care interventions have not been developed or adapted to meet the unique needs of culturally and linguistically diverse (CALD) communities [21]. This limits their effectiveness, as cultural factors significantly influence health beliefs and self-management behaviors [22].

Within Australia, the Indian community represents the second-largest immigrant community group, comprising over 721,000 people [23], and this population is at higher risk for poor CVD outcomes, including cardiovascular events, all-cause mortality, and hospitalizations [24]. Additionally, research indicates that more than half of the Indian immigrant population remains closely connected to their cultural values and belief systems, which shape their lifestyle choices and self-care decisions [25,26]. These cultural influences highlight the importance of involving individuals with lived experience of CVDs in the product design process to ensure the development of culturally relevant materials for their use [27].

To address this gap in culturally appropriate CA interventions, we have co-designed a culturally tailored CA intervention (Diaspora Engaged Self-Care Intervention and Heart [DESI-Heart]) to support self-care among individuals with CVD from the Indian diaspora in Australia. DESI-Heart integrates cultural elements specific to Indian communities—including language preferences, traditional health practices, and culturally congruent communication styles. In this protocol paper, we present a comprehensive overview of the co-designed DESI-Heart program, detailing its design and development process guided by established cultural adaptation frameworks. We also outline the planned pilot study to evaluate its feasibility and usability, and potential impact on self-care outcomes.

The primary aim was to evaluate the feasibility and usability of a co-designed culturally tailored CA (DESI-Heart) in supporting self-care management among Indian diaspora populations with CVDs in Australia over an 8-week intervention period.

The primary objectives were (1) to determine participant recruitment rates, retention rates, and adherence to the DESI-Heart program; and (2) to assess the usability of the DESI-Heart program using validated measures of the Bot Usability Scale.

The secondary objective was to evaluate preliminary effects of the DESI-Heart program on changes from baseline to 8 weeks in CVD self-care behaviors and quality of life.

## Methods

### Overview

The reporting and structure of the protocol were guided by the SPIRIT (Standard Protocol Items: Recommendations for Interventional Trials) 2025 statement and checklist [28] (Multimedia Appendix 1).

### Study Design

#### Theoretical Framework

**Figure 1:**
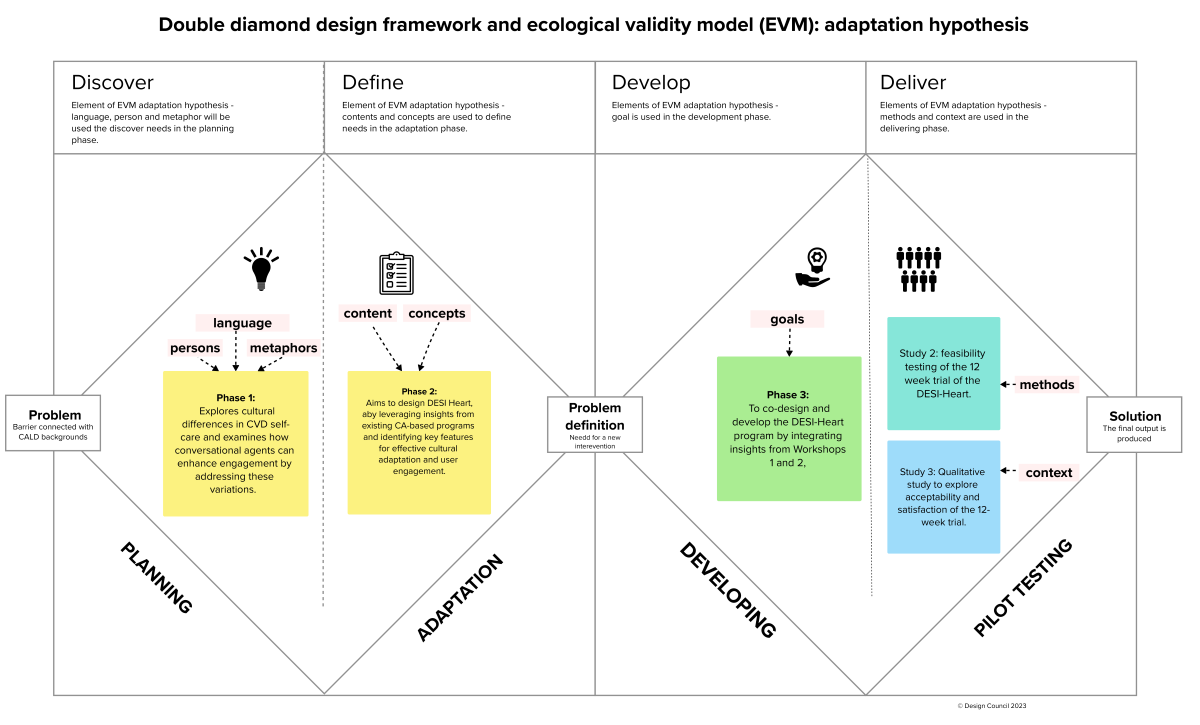
Logic flow of this study’s framework. CA: conversational agent; CALD: culturally and linguistically diverse; CVD: cardiovascular disease; DESI-Heart: Diaspora Engaged Self-Care Intervention and Heart; EVM: ecological validity model.

To inform the development of the DESI-Heart program, we used a combination of 2 theoretical frameworks: the Double Diamond Model (DDM) and the ecological validity model (EVM) [29]. The DDM focuses on providing a well-defined and comprehensive representation of design progress in developing novel interventions [30]. The DDM comprises 2 adjacent diamonds, and each diamond represents the process of exploring a problem broadly (divergent thinking) and the process of taking action to resolve the identified problem (convergent thinking) [31]. The DDM model has 4 processes distributed across these 2 adjacent diamonds. The first diamond is termed the problem phase, which consists of two major processes: (1) discover and (2) define. The second diamond is the solution phase, consisting of the other two processes, which are (3) develop and (4) deliver [32]. While the DDM provided the structured design approach, we integrated the EVM specifically to enhance cultural appropriateness and relevance. The EVM model has been used as an effective framework to guide culturally adapted interventions by increasing the ecological and external validity of the intervention [29]. To operationalize this framework, EVM encompasses eight cultural dimensions, which include (1) language used, (2) attributes of persons involved, (3) incorporation of metaphors, (4) identifying content, (5) understanding concepts, (6) achieving culturally adaptive intervention goals, (7) methods involved in intervention, and (8) context of the intervention [29,33]. To address the discover, define, and develop phases, we conducted a co-design study with end users—individuals from the Indian diaspora living with CVDs—using steps 1 to 6 of the EVM process. The deliver phase will involve developing a protocol to test the feasibility, usability, and acceptability of the DESI-Heart program, using steps 7 and 8 of the EVM process (Figure 1).

#### About CA

The intervention will be delivered using BuddyLink (Great Australian Pty Ltd), a research-grade, AI-enabled CA designed for developing and delivering interventions across various health care contexts [34]. BuddyLink is a validated platform for developing CAs, hosted in secure Australian data centers. The program relies on hard-coded, text- and logic-based conversations, with no AI or large language model principles underpinning its design. However, the current BuddyLink version uses AI in two assistive ways: (1) optional audio transcription or translation with transparent accuracy display, and (2) matching participant free-text inputs to predefined response options. BuddyLink has a proven record of secure, ethics-approved use across government, education, and health care projects, including health research at Deakin University [17,35].

#### Co-Design Results

**Figure 2:**
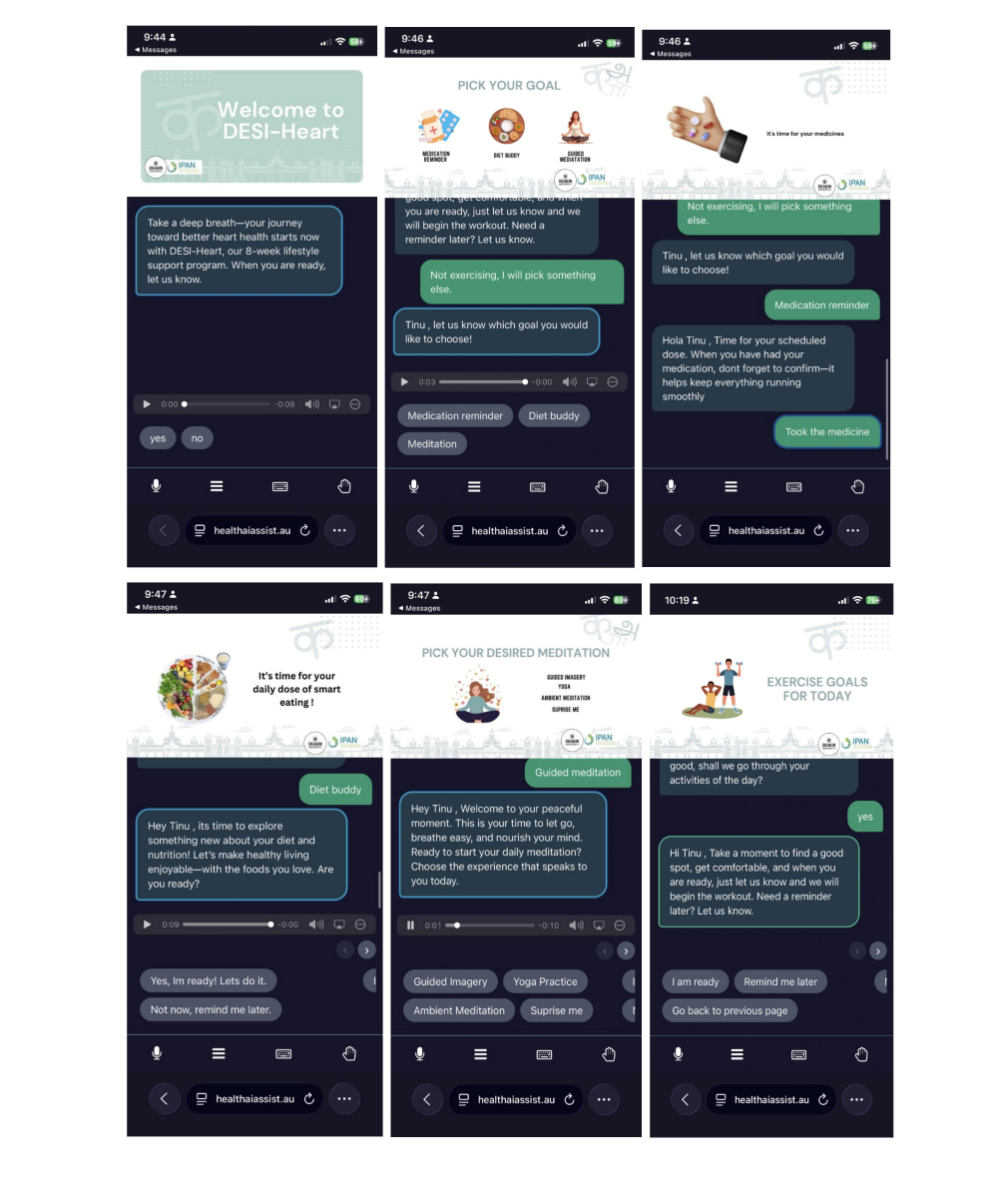
Screenshots of the DESI-Heart conversational agent. DESI-Heart: Diaspora Engaged Self-Care Intervention and Heart.

In DESI-Heart, 4 major goals or modules were identified through co-design workshops with end users: medication reminders, daily exercise activities, diet buddy support, and guided meditations. Each component was specifically developed to address key self-care challenges identified by the Indian diaspora living with CVD (Figure 2).

The medication reminders will support participants by providing timely alerts to take their medications. Additional features will include the ability to reschedule medication times, snooze reminders, and set alerts for medical appointments and diagnostic tests. Reminders will be scheduled according to the participant’s preferred times. If there are any changes in medication timing or if new medications are added during the program, participants can request updates in their program by informing the research team. The system does not prescribe medications, but only provides reminders, which will also be integrated with the participant’s Google Calendar, allowing them to track their medication schedule alongside other daily activities. However, participants will not have control over modifying this calendar, as it will function as a subscription calendar managed and updated by the research team.

The daily exercise component of the program is designed to support participants in meeting the Australian Physical Activity Guidelines, which recommend at least 150 minutes of moderate to vigorous physical activity per week, spread over 5 days or sessions [36]. To help achieve this, the DESI-Heart exercise program offers short, tailored workout sessions that include clear instructions and demonstrations, prerecorded by trained professionals. Participants will receive tailored exercise videos (≈2 minutes each) based on their preferred time of day. Each exercise will be delivered in 3 sets, scheduled across 5 days per week. Additionally, individuals with specific physical conditions will have access to condition-specific exercises to ensure the content remains safe, relevant, and appropriate for their needs. These exercise sessions are designed to resonate with the Indian community by incorporating culturally relevant content, speech, and delivery style. The demonstrations are led by trained professionals who reflect the participants’ cultural background. This approach was recommended by participants during the co-design workshops, as they expressed a desire for a sense of belongingness with a supportive, familiar presence during their engagement with the program.

Diet buddy is a nutrition education component designed to align with CVD-related dietary recommendations. This feature draws on resources from the recently updated Australian Dietary Guidelines and Australian Guide to Healthy Eating [37]. The content has been culturally adapted to reflect the diverse dietary habits commonly observed among Indian Australian communities. It incorporates both northern and southern Indian food practices alongside typical Australian food practices, ensuring that the program is relevant, inclusive, and accessible to participants from different regional and cultural backgrounds. Delivered gradually over 8 weeks, the program offers culturally tailored content on each food group based on the Australian Guide to Healthy Eating series, including hydration-related guidance and education on healthy sources of protein, fiber, and fats. It also includes convenient and budget-friendly meal and snack plans, meal plating strategies, structured meal plans, a recipe matrix, and practical guidance on incorporating phenolic compounds, polyphenols, and plant sterols to support healthier eating habits. Each week will focus on one of these components over the 8 weeks to ensure thorough education and help inform participants’ dietary practices.

Guided meditation is a key wellness component of the DESI-Heart program. Psychosocial stress is a commonly identified risk factor for CVD, and previous studies have shown that various forms of meditation and yoga can be effective in managing stress and improving CVD outcomes [38]. This component includes several modules, such as guided meditation, mindfulness breathing, and yoga, all of which are prerecorded with clear instructions and guidance from trained professionals. During the intervention period, participants will receive 1 meditation-based (≈10 minutes per meditation) session per day delivered via CA, over 8 weeks. The specific content, duration, and delivery times will be tailored to each participant’s requirements. As with other components of the program, these materials are designed to resonate with the Indian community by incorporating culturally relevant content, language, and delivery styles to foster more engagement and appropriateness of the content.

### Study Overview

An 8-week single-arm prepost pilot trial will be conducted. Participants will receive a personalized DESI-Heart program, which includes the 4 core modules described above, delivered via the CA at their preferred time of day. All assessments will be administered through web-based questionnaires at baseline and at the end of the 8-week program.

### Sample Size

Preliminary calculations using G-power indicated that a total of 28 participants will be sufficient to allow an (estimated) proportion of 80% for recruitment to be estimated with a 95% CI of ±15%.

### Participant and Recruitment

Participants will be adults (18 years and older) living with CVDs, including hypertension, heart failure, atrial fibrillation, coronary heart disease, or peripheral artery disease, and currently residing in Australia. Participants will also have to natively speak any Indian language apart from English; have access to the internet and a smartphone, tablet, or PC.

Participants will be excluded if they (1) have severe symptoms classified as New York Heart Association Class IV, a recent myocardial infarction or unstable angina, were referred to a cardiac transplant unit, or were in palliative care; (2) have severe chronic pulmonary disease; (3) live in a long-term care facility; (4) are unable to participate fully in this study for other reasons, including dementia, life-threatening comorbidities, or psychiatric disorders; and (5) did not identify themselves as part of any Indian community living in Australia. Participants will be recruited via social media (eg, Facebook [Meta]) and through Indian cultural associations, religious organizations, and community networks. To increase accessibility for less digitally literate or socially isolated individuals, we are also promoting this study via faith-based and community organizations, appealing to primary caregivers, and advertising at community gathering places.

### Procedure

After participants have provided informed consent, researchers will schedule a Zoom (Zoom Communications, Inc) or phone call based on participants’ preferences to complete the baseline data assessment. The baseline data assessment will include validated measures of self-care behaviors and quality of life, specifically: Medication Adherence Report Scale-10 [39], Godin Leisure-Time Exercise Questionnaire [40], Cardio-Med Food Frequency Survey Tool [41], and Self-Care Inventory [42]. During this session, participants will provide their sociodemographic details and medication history (Textbox 1).

**Feasibility and usability criteria and metrics.**

**Green**

- If one or more of the green criteria are met, this study will be considered feasible in its current form:
  At least ≥80% of the target sample size is recruited and enrolled in the Diaspora Engaged Self-Care Intervention and Heart (DESI-Heart) program within 3 months [35]
  At least ≥80% of participants remain in the DESI-Heart program [43]
  At least ≥66% of participants completed the set goals delivered via the DESI-Heart program [44]
  Attrition ≤20% [45]
- Conversational agent (CA) will be considered usable if the Bot Usability Scale score is >70 [46].

**Amber**

- If one or more of the amber criteria are met, this study will be considered likely feasible:
  50%-79% of the target sample recruited within 3 months
  60%-79% of participants remain in the DESI-Heart program
  50%-65% of participants completed the set goals delivered via the DESI-Heart program
  Attrition: 20.1%-35%
- CA will be considered moderately usable if the Bot Usability Scale score is between 51-69.

**Red**

- If one or more of the red criteria are met, this study will be considered not feasible in its current form:
  <50% of the target sample recruited within 3 months
  <60% of participants remain in this study at 8 weeks
  <50% of participants completed the set goals delivered via the DESI-Heart program
  Attrition >35%
- CA will be considered not usable if the Bot Usability Scale score is 50 or below.

Participants will complete a comprehensive online orientation conducted by the researcher, covering this study and its features as part of the DESI-Heart program. For medication reminders, participants will be asked to specify the times and durations for which they would like to receive notifications. In addition, they can opt to receive reminders for medication refills, upcoming general practitioner consultations, or scheduled diagnostic tests, if they choose to include these in their medication reminder goals. For physical activity and guided meditation, participants can select their preferred duration, schedule a time for meditation, and choose the intensity of exercise. For diet buddy, participants can select the time they prefer to receive the prompts and share relevant health information (age and conditions) to receive customized nutritional guidance tailored to their specific needs.

After completing the baseline questionnaires, the platform’s analytics system tracks user engagement metrics, including session frequency, duration, and completion patterns, to identify engagement barriers and inform future intervention refinements. By clicking the link, they will be directed to a web page where they can interact with the CA and access the program content. These links allow participants to access the intervention at any time. Upon accessing the platform, participants can select and complete goals of their choice, including revisiting missed goals or engaging with additional goals at the designated time. Participants will also have the option to choose their preferred language—English or one of several Indian languages (Hindi, Punjabi, Gujarati, Kashmiri, Bengali, Kannada, Tamil, Malayalam, and Telugu). Researchers will assist with the initial setup and help resolve any technical issues. Content will be delivered 4-5 times per day, 5 days a week, through prompts sent to participants’ mobile phones or emails at their preferred times. Each prompt will include a link to a specific goal-based dialogue with the CA on the BuddyLink platform.

For each self-care component, the system will deliver culturally tailored instructions and demonstrations followed by questions to determine whether participants completed the activity, their experience, and if they had any concerns. Participants’ responses to these questions will be recorded and saved to the BuddyLink database, enabling the research team to review weekly and modify content as required. During this period, researchers will check in via telephone weekly with participants to address any technical support needs.

**Figure 3:**
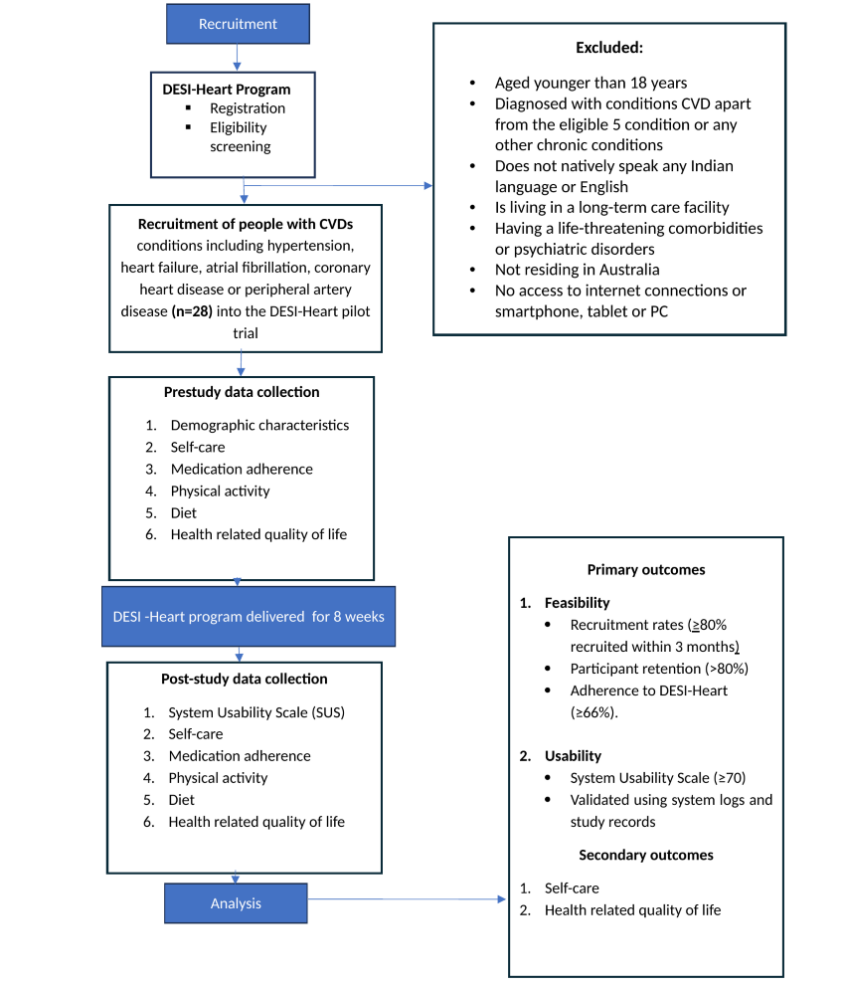
Overview of DESI-Heart study protocol. CVD: cardiovascular disease; DESI-Heart: Diaspora Engaged Self-Care Intervention and Heart.

After participants complete the 8-week DESI-Heart program, researchers will assist them in completing the follow-up post-study questionnaires online via Zoom. These will include all the questionnaires administered at baseline, minus the demographic questions. Additionally, participants will complete the Bot Usability Scale to report their perceived usability of the DESI-Heart program [46]. During pretesting, we observed that participants will be able to complete the full set of pre- and post-study questionnaires, including instructions, response time, and short breaks, within approximately 50 minutes. This supports the feasibility of administering the selected outcome measures without placing excessive burden on participants. Upon completion of the pilot study, participants will be remunerated for their time and involvement in the study (Figure 3). Subsequently, participants will also participate in an exit interview to better understand their perceptions of the DESI-Heart program, their level of satisfaction, and acceptability.

### User Testing

Before the commencement of this study, the platform was user-tested by research team members and volunteers over 2 weeks on both iOS (Apple Inc) and Android (Google LLC) operating systems. Feedback from this testing informed revisions to notification texts (via text messages and emails), improvements to navigation, adjustments to the user interface, and resolution of technical issues.

### Outcome Measures

The feasibility of this study will be measured using recruitment rates, participant retention, completion of the prescribed self-care program, and attrition. Feasibility will be assessed using criteria outlined in Textbox 1, informed by previous studies [35,43-45] and a “traffic light” system [47], to ensure transparent and standardized progression to a definitive trial. Usability will be assessed by the Bot Usability Scale [46]. Participant scores will be cross-validated with system logs and study records capturing interaction history, which will be accessible through BuddyLink. The usability of the DESI-Heart program is defined as a score of 70 and above, which corresponds to a percentile of 70%, indicating high perceived usability [46].

The secondary outcomes include changes (from baseline to 8 weeks) in participants’ self-care behaviors and quality of life as reported above (Table 1).

**Table 1:** Baseline and follow-up measures.

Assessment or procedure		Screening	Stage 1 (1 week)	Stage 2 (8 weeks)
**Procedures**				
	Informed consent	✓	✓	✓
	Demographic information	✓	✓	✓
	Medication Adherence Report Scale-10 [39]		✓	✓
	Godin Leisure-Time Exercise Questionnaire [40]		✓	✓
	Cardio-Med Food Frequency Survey Tool [41]		✓	✓
	Self-Care Inventory (SCI) [42]		✓	✓
**Assessments**				
	World Health Organization Quality of Life (WHOQOL) [48]		✓	✓
	Chatbot Usability Scale [46]			✓

### Data Analysis

For the primary outcomes (recruitment, retention, program completion, attrition, and usability), the analysis will focus on reporting the observed frequencies and rates. For secondary outcomes, changes in quantitative measures between baseline and 8-week follow-up will be analyzed using paired-sample *t* tests (2-tailed). Analyses will be based on the normality of the data, and if parametric assumptions are not met, data transformation will be considered to better satisfy these assumptions. All analyses will be conducted using Stata (Version SE V.17.0; StataCorp). Data will be stored in the Qualtrics secure firewall-protected server hosted by Deakin University. Subsequently, the data will be transferred to a Deakin-managed Syncplicity server, and the data will be permanently deleted from Qualtrics.

### Ethical Considerations

This study received ethics approval from the Royal Melbourne Hospital Human Research Ethics Committee (HREC/76317/MH-2021) for all procedures involving people living with CVDs. For this project, the platform operates as a dedicated instance with encrypted data storage, strict role-based access, and no local software requirements beyond a modern web browser. Personal identifiers or sensitive health data will not be collected; researchers can link participants only through nonidentifiable IDs, ensuring anonymity. Voice recordings are not stored, and transcriptions or logs can be deleted at any time upon request. End users retain full rights to access, correct, or delete their data via the platform’s privacy portal. Messages and email links sent to participants are delivered via the BuddyLink platform solely to provide access to the intervention. Upon completion of this study, participants’ mobile numbers and email addresses will be permanently deleted from the BuddyLink platform. The shared subscription calendar is independently hosted via Google Calendar. It will be sent to participants for download into their own calendars, and no information will be transferred to or shared via BuddyLink.

Before enrollment, all participants will be provided with a plain language statement outlining this study’s purpose, procedures, potential risks and benefits, the use of CAs and optional AI-enabled features, and how their data will be managed. Participants will be able to ask questions and receive clarification from the research team before deciding to participate. Informed consent will be obtained through an online consent form. Each participant will be provided with a AUD $100 (US $66.50) Woolworths Essentials gift card as compensation upon completion of the study. The datasets generated and analyzed during this study will be securely stored on the Deakin-managed Syncplicity server. These data will not be publicly available but may be made accessible by the primary investigator upon reasonable request and with approval from the Royal Melbourne Hospital Human Research Ethics Committee. To disseminate the findings, a summary of the results will be emailed to participants and presented to research teams. Findings will also be shared with the scientific community through peer-reviewed publications and conference presentations.

## Results

Recruitment for the pilot trial was scheduled to begin in June 2025 and concluded by September 2025. Participant follow-up will be completed by the end of December 2025. All 28 participants have now been recruited, and data analysis will be conducted once follow-up is finalized. Upon completion of data analysis, the findings from the pilot study will be submitted for publication.

## Discussion

### Principal Findings

This article outlines the protocol for pilot testing for the feasibility and usability of the DESI-Heart program, a systematically co-designed, culturally tailored CA designed specifically to support self-care management among Indian diaspora populations with CVDs in Australia. This innovative digital health solution addresses a critical gap in culturally appropriate self-care support for people living with CVDs belonging to different CALD backgrounds.

Through the advancement of natural language processing, AI, voice recognition, and large language models, CAs have become increasingly integrated into health care and health management [49]. These technologies offer scalable and cost-effective solutions that can provide medical support anytime through smartphones, mobile apps, or web-based platforms [50]. Evidence suggests that CA-based interventions can significantly support both patients and clinicians by assisting with specific tasks and processes [44]. Multiple CA-based interventions have demonstrated improvements in enhancing self-care behaviors, such as increased physical activity, healthier dietary habits, and better medication adherence [51]. However, much of the existing research and development has focused on the general population, with limited attention given to CA systems tailored for CALD communities [52]. Cultural beliefs and practices are deeply incorporated into everyday lives and affect how individuals approach self-care [22]. Due to these cultural differences, individuals from CALD communities often face additional challenges in navigating self-care. Their native cultural beliefs and practices may differ significantly from those of the host culture, leading to confusion, strain, or reduced engagement with mainstream health services [53,54]. This cultural disconnect can ultimately contribute to health disparities and poorer CVD outcomes among immigrant populations.

To address this gap, we applied DDM and EVM as cultural adaptation frameworks to guide the development and design of a culturally appropriate, CA-based CVD self-care management program. This methodologically rigorous approach ensured that cultural considerations were central rather than peripheral to the intervention design process. Through co-design workshops with end users, we identified key features to enhance the program’s cultural relevance, gathered ideas to improve cultural appropriateness, and received suggestions to increase participant engagement with the culturally appropriate self-care intervention.

DESI-Heart program—includes 4 carefully designed core modules (medication reminders, physical activity support, dietary guidance, and stress management through meditation). The content has been specifically developed to resonate with the Indian community by incorporating culturally relevant examples, familiar language patterns, and delivery styles that align with cultural norms, in order to foster engagement and enhance content appropriateness. This program also provides access to DESI-Heart in multiple Indian languages, increasing the accessibility of support to those who speak languages other than English.

By providing culturally and linguistically appropriate self-care information and support, the DESI-Heart program aims to increase engagement in self-care behaviors that are culturally meaningful and sustainable. The CA used in this study has been designed to be user-friendly across varying levels of digital literacy, addressing a known barrier to digital health adoption among older adults and immigrant populations. It incorporates advanced voice recognition technology capable of understanding diverse accents, dialects, and several Indian languages, ensuring accessibility for a broader range of participants. Participants can interact with the CA either through voice commands or touch, allowing flexible access to care based on their preference. This makes DESI-Heart a more inclusive and accessible CA-delivered CVD self-care management tool, particularly when compared to existing CA interventions that often lack cultural tailoring [15,52]. Moreover, DESI-Heart supports participants in integrating their own cultural beliefs with key aspects of the host country’s health care norms. This dual approach not only enhances the effectiveness of the intervention for CALD communities but also positions DESI-Heart as a model for culturally responsive digital health support.

Despite its innovative features, several important limitations of the DESI-Heart program warrant acknowledgment. While the program was designed based on general Indian cultural values, it did not fully account for the regional and linguistic diversity that exists within the Indian community, particularly the differences between northern and southern cultural practices. These regional variations can significantly influence health-related behaviors, beliefs, and preferences [55], which may affect the program’s relevance and effectiveness for some participants. Additionally, as a pilot feasibility study with a relatively small sample size and single-arm design, this investigation cannot definitively evaluate efficacy outcomes. The current inclusion criteria limit the program’s inclusivity, as participants with severe conditions or those residing in care facilities were not included, which may affect the interpretation and applicability of the findings.

Findings from this pilot study will offer critical insights to guide the design of a future, fully powered efficacy trial. These will include broader eligibility criteria, refined recruitment strategies, enhancements to CA-related features and functions to improve engagement, and tailored delivery preferences for specific participant subgroups, thereby strengthening the generalizability of the results. Future studies could also focus on specific ethnic groups with similar cultural and linguistic backgrounds to provide more targeted and appropriate support. Additionally, trial findings may guide the implementation and adaptation of this approach for CVD management services within other CALD communities, supporting their specific cultural needs and requirements while making health care more accessible, appropriate, and equitable. The knowledge generated may also contribute to a broader understanding of how to effectively incorporate cultural elements into digital health interventions.

### Conclusions

The DESI-Heart program is a culturally and linguistically tailored self-care intervention designed to support Indian adults living with CVD in Australia. Through co-design workshops, end users identified 4 key goals to enhance self-care engagement by incorporating culturally relevant activities, knowledge, and content aligned with their cultural identity and linguistic preferences. This protocol outlines a pilot study to assess the feasibility and usability of the program, which will inform the design of a larger evaluation trial. Based on insights gained from this pilot study, future studies will incorporate comparator groups to rigorously assess the program’s efficacy. This approach has the potential to strengthen existing health care services by providing more inclusive and accessible support for CALD populations.

## Appendix

Multimedia Appendix 1 SPIRIT 2025 checklist: recommended items to address in a clinical trial protocol and related documents.

## Abbreviations

AI: artificial intelligence
CA: conversational agent
CALD: culturally and linguistically diverse
CVD: cardiovascular disease
DDM: Double Diamond Model
DESI-Heart: Diaspora Engaged Self-Care Intervention and Heart
EVM: ecological validity model
SPIRIT: Standard Protocol Items: Recommendations for Interventional Trials

## References

[1] Mensah GA, Roth GA, Fuster V (2019). The global burden of cardiovascular diseases and risk factors: 2020 and beyond. J Am Coll Cardiol.

[2] Marquina C, Talic S, Vargas-Torres S, Petrova M, Abushanab D, Owen A, Lybrand S, Thomson D, Liew D, Zomer E, Ademi Z (2022). Future burden of cardiovascular disease in Australia: impact on health and economic outcomes between 2020 and 2029. Eur J Prev Cardiol.

[3] (2013). Global action plan for the prevention and control of noncommunicable diseases 2013-2020. World Health Organization.

[4] Riegel B, Moser DK, Buck HG, Dickson VV, Dunbar SB, Lee CS, Lennie TA, Lindenfeld J, Mitchell JE, Treat-Jacobson DJ, Webber DE, American Heart Association Council on CardiovascularStroke Nursing; Council on Peripheral Vascular Disease;Council on Quality of CareOutcomes Research (2017). Self-care for the prevention and management of cardiovascular disease and stroke: a scientific statement for healthcare professionals from the American Heart Association. J Am Heart Assoc.

[5] Zeng L, Xu X, Perry L (2025). Self-care behaviours of first-generation Chinese immigrants living with cardiovascular disease: a qualitative study. J Adv Nurs.

[6] Kessing D, Denollet J, Widdershoven J, Kupper N (2016). Psychological determinants of heart failure self-care: systematic review and meta-analysis. Biopsychosoc Sci Med.

[7] Zhang X, Ma H, Lam C, Ho G, Mak Y (2023). Effectiveness of acceptance and commitment therapy on self-care, psychological symptoms, and quality of life in patients with cardiovascular disease: a systematic review and meta-analysis. J Contextual Behav Sci.

[8] Pagoto S, Bennett GG (2013). How behavioral science can advance digital health. Transl Behav Med.

[9] Alhussein G, Hadjileontiadis L (2022). Digital health technologies for long-term self-management of osteoporosis: systematic review and meta-analysis. JMIR mHealth uHealth.

[10] Ware P, Bartlett SJ, Paré G, Symeonidis I, Tannenbaum C, Bartlett G, Poissant L, Ahmed S (2017). Using eHealth technologies: interests, preferences, and concerns of older adults. Interact J Med Res.

[11] Xesfingi S, Vozikis A (2016). eHealth literacy: in the quest of the contributing factors. Interact J Med Res.

[12] Torrent-Sellens J, Díaz-Chao Á, Soler-Ramos I, Saigí-Rubió F (2016). Modelling and predicting ehealth usage in Europe: a multidimensional approach from an online survey of 13,000 European union internet users. J Med Internet Res.

[13] Siren A, Knudsen SG (2017). Older adults and emerging digital service delivery: a mixed methods study on information and communications technology use, skills, and attitudes. J Aging Soc Policy.

[14] Kim BY, Lee J (2017). Smart devices for older adults managing chronic disease: a scoping review. JMIR mHealth uHealth.

[15] Moulik S, Chatterjee S (2021). DIL - a proof of concept study to show the efficacy of conversational agents for heart failure patients. JSIS.

[16] McTear MF, Callejas Z, Griol D (2016). The Conversational Interface.

[17] Jansons P, Robins L, O'Brien L, Haines T (2017). Gym-based exercise and home-based exercise with telephone support have similar outcomes when used as maintenance programs in adults with chronic health conditions: a randomised trial. J Physiother.

[18] Nishida T, Nakazawa A, Ohmoto Y, Mohammad Y (2014). Conversational Informatics: A Data-Intensive Approach With Emphasis on Nonverbal Communication.

[19] Gong E, Baptista S, Russell A, Scuffham P, Riddell M, Speight J, Bird D, Williams E, Lotfaliany M, Oldenburg B (2020). My Diabetes Coach, a mobile app-based interactive conversational agent to support type 2 diabetes self-management: randomized effectiveness-implementation trial. J Med Internet Res.

[20] Wolters MK, Kelly F, Kilgour J (2016). Designing a spoken dialogue interface to an intelligent cognitive assistant for people with dementia. Health Inf J.

[21] Whitehead L, Talevski J, Fatehi F, Beauchamp A (2023). Barriers to and facilitators of digital health among culturally and linguistically diverse populations: qualitative systematic review. J Med Internet Res.

[22] Karimi M, Clark AM (2016). How do patients' values influence heart failure self-care decision-making?: A mixed-methods systematic review. Int J Nurs Stud.

[23] (2022). Cultural diversity: census: information on country of birth, year of arrival, ancestry, language and religion. ABS.

[24] (2021). People in Australia who were born in India: 2021 census country of birth QuickStats. ABS.

[25] Payyappallimana U (2013). Health and well-being in Indian local health traditions. An Integrated View of Health and Well-Being: Bridging Indian and Western Knowledge.

[26] Kamath DY, Bhuvana KB, Salazar LJ, Varghese K, Kamath A, Idiculla J, Pais P, Kulkarni S, Granger BB, Xavier D (2021). A qualitative, grounded theory exploration of the determinants of self-care behavior among Indian patients with a lived experience of chronic heart failure. PLoS One.

[27] Stamp KD, Prasun M, Lee CS, Jaarsma T, Piano MR, Albert NM (2018). Nursing research in heart failure care: a position statement of the American Association of Heart Failure Nurses (AAHFN). Heart Lung.

[28] Chan A, Boutron I, Hopewell S, Moher D, Schulz K, Collins G, Tunn R, Aggarwal R, Berkwits M, Berlin JA, Bhandari N, Butcher NJ, Campbell MK, Chidebe RC W, Elbourne DR, Farmer AJ, Fergusson DA, Golub RM, Goodman SN, Hoffmann TC, Ioannidis JP A, Kahan BC, Knowles RL, Lamb SE, Lewis S, Loder E, Offringa M, Ravaud P, Richards DP, Rockhold FW, Schriger DL, Siegfried NL, Staniszewska S, Taylor RS, Thabane L, Torgerson DJ, Vohra S, White IR, Hróbjartsson A (2025). SPIRIT 2025 statement: updated guideline for protocols of randomised trials. BMJ.

[29] Bernal G, Bonilla J, Bellido C (1995). Ecological validity and cultural sensitivity for outcome research: issues for the cultural adaptation and development of psychosocial treatments with Hispanics. J Abnorm Child Psychol.

[30] (2004). Framework for innovation. DesignCouncil.

[31] (2015). The design process; what is the double diamond?. DesignCouncil.

[32] Liu D, Hsu HF (2009). An international comparison of empirical generalized double diamond model approaches to Taiwan and Korea. Competitiveness Rev: Int Bus J.

[33] Magaña S, Lopez K, Aguinaga A, Morton H (2013). Access to diagnosis and treatment services among Latino children with autism spectrum disorders. Intellect Dev Disabil.

[34] Gvozdenko E (2025). BuddyLink - AI-enabled applications in health, aged care and education. Great Australian Pty Ltd.

[35] Maddison R, Nourse R, Daryabeygikhotbehsara R, Tegegne TK, Jansons P, Rawstorn JC, Atherton J, Driscoll A, Oldenburg B, Vasa R, Kostakos V, Dingler T, Abbott G, Scuffham P, Manski-Nankervis JE, Kwasnicka D, Kensing F, Islam SMS, Maeder A, Zhang Y (2025). Digital home-based self-monitoring system for people with heart failure: protocol for development of SmartHeart and evaluation of feasibility and acceptability. JMIR Res Protoc.

[36] Bauman AE, Nau T, Cassidy S, Gilbert S, Bellew W, Smith BJ (2021). Physical activity surveillance in Australia: standardisation is overdue. Aust N Z J Public Health.

[37] (2013). Australian dietary guidelines. National Health and Medical Research Council.

[38] Schneider RH, Grim CE, Rainforth MV, Kotchen T, Nidich SI, Gaylord-King C, Salerno JW, Kotchen JM, Alexander CN (2012). Stress reduction in the secondary prevention of cardiovascular disease: randomized, controlled trial of transcendental meditation and health education in Blacks. Circ Cardiovasc Qual Outcomes.

[39] Chan AHY, Horne R, Hankins M, Chisari C (2020). The medication adherence report scale: a measurement tool for eliciting patients' reports of nonadherence. Br J Clin Pharmacol.

[40] Godin G (2011). The Godin-Shephard leisure-time physical activity questionnaire. Health Fitness J Canada.

[41] Kucianski T, Thodis A, Vally H, Kouris-Blazos A, Moschonis G, Wilson A, van Gaal W, Tierney A, Itsiopoulos C (2020). The cardio-med survey tool: development and pilot validation of a FFQ in a multicultural cardiology cohort. Public Health Nutr.

[42] Luciani M, De Maria M, Page SD, Barbaranelli C, Ausili D, Riegel B (2022). Measuring self-care in the general adult population: development and psychometric testing of the self-care inventory. BMC Public Health.

[43] Kelders SM, Kok RN, Ossebaard HC, Van Gemert-Pijnen JE (2012). Persuasive system design does matter: a systematic review of adherence to web-based interventions. J Med Internet Res.

[44] Griffin A, Xing Z, Khairat S, Wang Y, Bailey S, Arguello J, Chung A (2021). Conversational agents for chronic disease self-management: a systematic review. AMIA Annu Symp Proc.

[45] Nunan D, Aronson J, Bankhead C (2018). Catalogue of bias: attrition bias. BMJ Evidence-Based Med.

[46] Borsci S, Malizia A, Schmettow M, van der Velde F, Tariverdiyeva G, Balaji D, Chamberlain A (2022). The chatbot usability scale: the design and pilot of a usability scale for interaction with AI-based conversational agents. Pers Ubiquit Comput.

[47] Lewis M, Bromley K, Sutton CJ, McCray G, Myers HL, Lancaster GA (2021). Determining sample size for progression criteria for pragmatic pilot RCTs: the hypothesis test strikes back!. Pilot Feasibility Stud.

[48] Kim S (2023). World Health Organization Quality of Life (WHOQOL) assessment. Encyclopedia of Quality of Life and Well-Being Research.

[49] Kramer LL, Ter Stal S, Mulder BC, de Vet E, van Velsen L (2020). Developing embodied conversational agents for coaching people in a healthy lifestyle: scoping review. J Med Internet Res.

[50] Bickmore TW, Kimani E, Trinh H, Pusateri A, Paasche-Orlow M, Magnani J (2018). Managing chronic conditions with a smartphone-based conversational virtual agent.

[51] Singh B, Olds T, Brinsley J, Dumuid D, Virgara R, Matricciani L, Watson A, Szeto K, Eglitis E, Miatke A, Simpson CEM, Vandelanotte C, Maher C (2023). Systematic review and meta-analysis of the effectiveness of chatbots on lifestyle behaviours. NPJ Digit Med.

[52] Bin Sawad A, Narayan B, Alnefaie A, Maqbool A, Mckie I, Smith J, Yuksel B, Puthal D, Prasad M, Kocaballi AB (2022). A systematic review on healthcare artificial intelligent conversational agents for chronic conditions. Sensors (Basel).

[53] Osokpo O, Riegel B (2021). Cultural factors influencing self-care by persons with cardiovascular disease: an integrative review. Int J Nurs Stud.

[54] Osokpo OH, James R, Riegel B (2021). Maintaining cultural identity: a systematic mixed studies review of cultural influences on the self-care of African immigrants living with non-communicable disease. J Adv Nurs.

[55] Dutt AK, Noble AG (1982). The culture of India in spatial perspective: an introduction. India: Cultural Patterns and Processes.

